# Demographic variables, anthropometric indices, sleep quality, Metabolic Equivalent Task (MET), and developing diabetes in the southwest of Iran

**DOI:** 10.3389/fpubh.2023.1020112

**Published:** 2023-03-14

**Authors:** Seyed Ahmad Hosseini, Samira Beiranvand, Kourosh Zarea, Kourosh Noemani

**Affiliations:** ^1^Nutrition and Metabolic Diseases Research Center, Clinical Research Institute, Ahvaz Jundishapur University of Medical Sciences, Ahvaz, Iran; ^2^Nursing Department, Nursing Care Research Center in Chronic Diseases, Nursing and Midwifery School, Ahvaz Jundishapur University of Medical Sciences, Ahvaz, Iran; ^3^Department of Disease Prevention and Control, Deputy of Health Center, Ahvaz Jundishapur University of Medical Sciences, Ahvaz, Iran

**Keywords:** demographic factors, sleep quality, metabolic equivalent task, diabetes, anthropometric indices, Persian cohort, Hoveyzeh

## Abstract

**Propose:**

The present study has sought to investigate the prevalence of diabetes and its related risk factors, to examine the relationship between demographic variables, anthropometric indices, sleep quality, and Metabolic Equivalent Task (MET) with diabetes in Khuzestan province, southwest Iran.

**Methods:**

The present study has a cross-sectional design (the baseline data of the Hoveyzeh cohort study as a sub-branch of the Persian Prospective Cohort Study). Comprehensive information from 10,009 adults (aged 35–70 years) was collected from May 2016 to August 2018 through a multi-part general questionnaire containing general characteristics, marital status, education, smoking, sleep quality, MET, and anthropometric indices. Data analysis was performed by SPSS software version 19.

**Results:**

The mean age of the sample was 52.97 ± 8.99 years. 60.3% of the population were women and 67.7% were illiterate. Out of the 10,009 people surveyed, 1,733 stated that they have diabetes (17%). In 1,711 patients (17%) the amount of FBS was ≥126 mg/dl. There is a statistically significant relationship between diabetes and MET. More than 40% had BMI above 30. Anthropometric indices in diabetic and non-diabetic individuals were different. Also, there was a statistically significant difference between the mean duration of sleep and the use of sleeping pills in diabetic and non-diabetic groups (*p* < 0.05). Based on logistic regression, marital status [OR = 1.69 (95% CI, 1.24, 2.30)], education level [OR = 1.49 (95% CI, 1.22, 1.83)], MET [OR = 2.30 (95% CI, 2.01, 2.63)], height [OR = 0.99 (95% CI, 0.98, 0.99)], weight [OR = 1.007 (95% CI, 1.006, 1.012)], wrist circumference [OR = 1.10 (95% CI, 1.06, 1.14)], waist circumference [OR = 1.03 (95% CI, 1.02, 1.03)], waist-to-hip ratio [OR = 3.41 (95% CI, 2.70, 4.29)], and BMI [OR = 2.55 (95% CI, 1.53, 4.25)], are good predictors for diabetes.

**Conclusion:**

The results of this study showed that the prevalence of diabetes in Hoveyzeh city, Khuzestan, Iran, was almost high. and emphasize that preventive interventions should focus on risk factors, especially socioeconomic status, and anthropometric indicators along with lifestyle.

## Background

Among the Eastern Mediterranean Region (EMR) countries, Iran is the second-most populous country after Pakistan and is expected to face a large increase in the prevalence of diabetes by 2030. According to statistics the prevalence of diabetes and related risk factors in Iran was 10.9% (1). Diabetes is considered a serious threat to global health, which is associated with a sedentary lifestyle and excessive calorie intake, and stress. The World Health Organization (WHO) has declared diabetes to be a latent epidemic (2). Prevention at different levels can reduce the complications and incidence of this disease, especially at the initial stages. So, identifying and controlling its risk factors is important (3). Ting et al. stated that recognizing the early markers for predicting diabetes and implementing related prevention strategies are among the critical steps to mitigate this problem worldwide (4).

Based on the literature, there is a strong association between demographic variables, lifestyle variables, clinical variables, and future diabetes. The risk of developing diabetes increases if there are some risk factors such as positive family history, old age, obesity, and lack of physical activity (4–6). It has also been shown that ethnic and social differences lead to different prevalences of diabetes (7). Ramezankhani et al. stated that the prevalence of diabetes is related to some demographic and social factors such as gender, urbanization, level of education, marital status, and occupation (8). Besides, Rahimian et al. reported that the prevalence of diabetes was only related to education level and not to gender and marital status (9).

On the other hand, one of the important and independent risk factors for diabetes is obesity, which is measured by various anthropometric indices such as waist circumference, hip circumference, and body mass (10, 11). Some studies have also shown that four anthropometric indices of Body Mass Index (BMI), Waist Circumference (WC), Waist-to-Hip Ratio (WHR), and Waist-to-Height Ratio (WHtR), as obesity indicators which can be used to estimate the risk of diabetes in the future. It should be noted that the emphasis of studies on each of the above indicators in the forecasts has been different and some other indicators have been proposed along with these four main indicators (4, 12–15).

As a new risk factor, sleep disorders also play an important role in the development of diabetes through the neuro-metabolic pathway. Following sleep deprivation, cortisol levels rise and inhibit insulin production, and may lead to a diabetic or pre-diabetic condition in the long run. In addition, following sleep disorders, insulin sensitivity decreases and an increase in blood glucose levels leads to a decrease in the quality and quantity of sleep, which in turn leads to the progression of diabetes (16, 17). The results of studies in this field also confirm the relationship between sleep quality and the incidence of diabetes. However, in this regard, there are differences in the results obtained from different populations (11, 18, 19).

Moreover, many studies have examined the relationship between physical activity and diabetes. In a systematic review and meta-analysis, Aune et al. reviewed 87 studies in this area and found that most studies indicated that there was a significant inverse relationship between different types of physical activity and the incidence of diabetes. In addition, the intensity of activity should be moderate to severe and every week (5–7 h per week). However, the reduction of accumulated fat in the body in this case plays a mediating role (20).

According to the recommendation of the International Diabetes Federation (IDF), one of the ways to reduce the impact of diabetes at the local, regional, and global levels is to improve the quality of epidemiological research on diabetes by strengthening surveys and regular monitoring systems (21). Although the general risk factors used in disease risk calculations are fixed and reliable in different populations, they are not ideal in different ethnicities and diseases such as diabetes due to the presence of some specific or different risk factors (22). Besides, in a situation where up-to-date data from a national information system are not yet available, population-based studies must be conducted periodically to obtain new information on the country's healthcare needs (23). In this regard, prospective cohort studies can be an ideal choice for examining multiple outcomes, different and simultaneous risk factors for non-communicable diseases, examining the relationships between them, and designing preventive strategies by policymakers (24, 25). Therefore, considering the lack of comprehensive and up-to-date information on the prevalence of diabetes and related risk factors in Khuzestan province, the present study has sought to investigate the prevalence of diabetes and its related risk factors, to examine the relationship between demographic variables, anthropometric indices, sleep, and physical activity with diabetes in Khuzestan province.

## Method

The present study was a cross-sectional study that was performed to determine the risk factors for diabetes in Khuzestan, southwest Iran. This study is a part of the Hoveyzeh Cohort Study (HCS) which is also a sub-branch of the Persian cohort study (26). Comprehensive information on the design and baseline characteristics of the HCS was published in 2020 (22). In this population-based study, 10,009 adults (aged 35–70 years) were recruited from May 2016 to August 2018. The eligible individuals were included from both sexes. They lived in urban and rural areas. Those who were unable to communicate or respond, and individuals with mental disorders, intellectual disabilities, and any psychiatric illness (e.g., psychosis) in an acute stage who were under medical treatment, were excluded from the study. Informed consent was obtained from all participants, and they were enrolled using their national ID cards. According to a standard protocol (22), blood and urine samples from subjects were taken by trained laboratory staff, and immediately after that, anthropometric data (weight, height, waist, hip, and wrist circumferences, and blood pressure) were also measured. BMI and WHR were calculated based on anthropometric data. In the third stage, general data were collected through interviews with individuals or, if necessary, with their relatives. This questionnaire included general demographics (gender, age, marital status, and education), 2 questions about alcohol and tobacco use, 5 questions about sleep (amount of sleep, night work, use of sleeping pills), and one question with 28 items about MET (duration and intensity of physical activity, daily activities, exercise). Follow-ups began 1 year after the first day of enrollment and data entry and they continued annually by telephone. Data analysis was performed by SPSS software version 19. Data were presented as (mean ± standard deviation) and frequency (%) for quantitative and qualitative variables, respectively. An independent *t*-test was used to compare the values of variables between men and women. Pearson correlation coefficient was used to determine the correlation between each of the anthropometric indices and risk factors for diabetes. *P* < 0.05 were considered statistically significant. The individuals with incomplete data and those who did not take part in different stages of follow-up were excluded from the analysis. The study was approved by the Ethics Committee of Ahvaz University of Medical Sciences.

## Results

The mean age of the diabetic subjects was 52.97 ± 8 8.99 years. More than 60% of the population were women (60.3%) and illiterate (67.7%). Out of the 10,009 people surveyed, 1,733 people stated that they have diabetes (17.3%). It was also found that in 1,711 patients (17%) the amount of FBS was ≥126 mg/dl. Two thousand three hundred and eleven of the subjects (20.94%) had FBS = 100–125 mg/dl and were classified as pre-diabetic. A total of 2,226 people (based on self-report and FBS check) were diagnosed with diabetes. The prevalence of diabetes in the studied population was 22.2%. In addition, the results of the Chi-square test showed that there is a statistically significant relationship between diabetes and MET. Other demographic and lifestyle information is provided in Table 1.

**Table 1:** Relationship between demographic variables and diabetes (*n* = 10,009).

**Variables**			**Frequency**	**Percent**	*P*-value^*^
Gender	Female		1,343	60.3	0.54
	Male		883	39.7	
Age (Mean ± SD)	Diabetes		52.97	8.99	0.000
	Non-diabetes		47.72	8.99	
Marital status	Single		48	2.2	0.000
	Married		1,893	85	
	Widow		253	11.4	
	Divorced		32	1.4	
Education status	Illiterate		1,507	67.7	0.000
	Primary school		327	14.7	
	Secondary school		113	5.1	
	High school diploma		152	6.8	
	University		127	5.7	
Smoking	User		492	22.1	0.137
	Non-user		1,734	77.9	
Metabolic equivalent task (MET)	1^st^ quarter	Diabetes	784	31.3	0.000
		Non-diabetes	1,719	68.7	
	2^nd^ quarter	Diabetes	557	22.2	
		Non-diabetes	1,948	77.8	
	3^rd^ quarter	Diabetes	473	18.9	
		Non-diabetes	2,035	81.1	
	4^th^ quarter	Diabetes	412	16.5	
		Non-diabetes	2,081	83.5	

* Comparison of diabetic and non-diabetic groups.

More than 17% of people had a normal BMI (18.5–24.9) and 43.4% had BMI above 30. The results of the independent *t*-test showed that there is a significant difference between the mean of anthropometric indices (weight, waist circumference, wrist circumference, and height) in diabetic and non-diabetic individuals. Information related to anthropometric variables is given in Table 2.

**Table 2:** Relationship between anthropometric indices and diabetes (*n* = 10,009).

**Anthropometric indicators**		**Mean**	**SD**	***P*-value**
Weight (Kg)	Diabetes	79.68	15.41	0.000
	Non-diabetes	77.59	15.58	
Height (cm)	Diabetes	164.06	8.94	0.003
	Nondiabetes	164.70	9.16	
Hip Circumference	Diabetes	104.10	10.28	0.816
	Non-diabetes	104.04	9.59	
Wrist Circumference	Diabetes	17.59	1.35	0.000
	Nondiabetes	17.42	1.33	
Waist circumference	Diabetes	103.05	11.62	0.000
	Non-diabetes	98.68	12.05	
Waist to hip ratio (WHR)	Diabetes	0.99	0.063	0.000
	Non-diabetes	0.94	0.064	
Body mass index (BMI)	Underweight ( ≤ 18.5)	0.8	17	0.000
	Normal (18.5–24.9)	17.4	384	
	Overweight (25–25.9)	38.6	859	
	Obese (≥30)	43.4	966	

The mean duration of sleep in diabetic patients was 7.69 ± 1.60 and 31.5% of people used sleeping pills. The results of the independent *t*-test and chi-square test showed that there was a statistically significant difference between the mean duration of sleep and the use of sleeping pills in diabetic and non-diabetic groups (*p* < 0.05). Other information about sleep is given in Table 3.

**Table 3:** Relationship between sleep-related variables and diabetes (*n* = 10,009).

**Variables**			**Frequency**	**Percent**	***P*-value**
Leg restlessness	Yes	Diabetes	391	24.9	0.010
		Non-diabetes	1,178	75.1	
	No	Diabetes	1,568	21.5	
		Non-diabetes	5,717	78.5	
	Don't Know	Diabetes	267	23.1	
		Non-diabetes	888	76.9	
Sleeping pills use	Non-user	User	2,144	22	0.000
		Non-user	7,605	78	
	User	User	82	31.5	
		Non-user	178	68.5	
Sleep duration (Mean ± SD)	Diabetes		7.61	1.53	0.021
	Non-diabetes		7.69	1.60	

A logistic regression test was used to determine the important predictors of diabetes. According to the regression coefficients, there exists a significant relationship between diabetes and marital status [OR = 1.69 (95% CI, 1.24, 2.30)], an education level [OR = 1.49 (95% CI, 1.22, 1.83)], MET [OR = 2.30 (95% CI, 2.01, 2.63)], height [OR = 0.99 (95% CI, 0.98, 0.99)], weight [OR = 1.007 (95% CI, 1.006, 1.012)], wrist circumference [OR = 1.10 (95% CI, 1.06, 1.14)], waist circumference [OR = 1.03 (95% CI, 1.02, 1.03)], waist-to-hip ratio [OR = 3.41 (95% CI, 2.70, 4.2)], BMI [OR = 2.55 (95% CI, 1.53, 4.25)], are good predictors for diabetes. Considering that the significant values mentioned for these variables are < 0.05, it can be said that these variables are a good predictor for the dependent variable of diabetes (Table 4).

**Table 4:** Univariate raw logistic regression in relation to diabetes (*n* = 10,009).

**Parameter**	**Crude odds ratio**	**SE**	**95% CI for OR**	***P***
**Gender**				
Male	Ref	-	-	-
Female	1.030	0.049	[9.36, 1.13]	0.544
**Marital status**				
Single	Ref	-	-	-
Married	1.694	0.158	[1.24, 2.30]	0.001
Widow	3.213	0.174	[2.28, 4.51]	0.000
Divorced	1.436	0.251	[0.87, 2.34]	0.149
**Educational status**				
Academic	Ref	-	-	-
Illiterate	1.499	0.102	[1.227, 1.831]	0.000
Primary school	1.143	0.116	[0.911, 1.434]	0.247
Secondary school	0.944	0.142	[0.714, 1.247]	0.684
High school diploma	1.207	0.134	[0.929, 1.568]	0.159
**Smoking**				
Non-user	Ref	-	-	-
User	1.090	0.058	[0.973, 1.222]	0.137
**BMI**				
Underweight ( ≤ 18.5)	Ref	-	-	-
Normal (18.5–24.9)	1.604	0.264	[0.957, 2.689]	0.073
Overweight (25–25.9)	2.338	0.261	[1.403, 3.896]	0.001
Obese (≥30)	2.552	0.260	[1.532, 4.251]	0.000
**MET**				
4^th^ quarter	Ref	-	-	-
1^st^ quarter	2.304	0.069	[2.012, 2.637]	0.000
2^nd^ quarter	1.444	0.072	[1.254, 1.664]	0.000
3^rd^ quarter	1.174	0.074	[1.015, 1.358]	0.031
**WHR**				
Normal	Ref	-	-	-
Abnormal	3.410	0.118	[2.706, 4.297]	0.000
**Sleep pill use**				
Non-user	Ref	-	-	-
User	1.634	0.136	[1.252, 2.132]	0.000
**Leg restlessness**				
Don't know	Ref	-	-	-
Yes	1.104	0.091	[0.924, 1.319]	0.277
No	0.912	0.075	[0.787, 1.057]	0.223
Age real	1.062	0.003	[1.05, 1.06]	0.000
Weight (kg)	1.009	0.002	[1.006, 1.012]	0.000
Waist circumference	1.031	0.002	[1.026, 1.035]	0.000
Hip circumference	1.001	0.002	[0.996, 1.005]	0.812
Wrist circumference	1.101	0.018	[1.064, 1.140]	0.000
Height (cm)	0.992	0.003	[0.987, 0.997]	0.003
Sleep duration	1.036	0.016	[1.005, 1.068]	0.021

## Discussion

Based on our findings, the percentage of diabetes in the population aged 35–70 years in Hoveyzeh city, Khuzestan, Iran, was quite above 20%. Our results were lower than those reported in some previous studies (5, 27) and higher than in some other studies (3, 28). Considering the smaller geographical scale and the smaller target population, our results are significant, indicating the need for proper planning, early interventions, and lifestyle change. These differences can be attributed to exposure to risk factors for diabetes, urbanization, poor diet, obesity, and genetic structure of individuals (29, 30).

Our results also showed that some demographic variables are associated with the risk of diabetes, so with increasing age, the chance of diabetes increases 1.062 times. In this regard, Akbarzadeh et al. reported that every 5 years of aging, the risk of diabetes increases by 18% (28). Kivimaki et al. reported that people aged 55–59 were almost twice as likely to develop diabetes as those aged 35–39 (31). Based on the results of previous studies, aging is associated with chronic inflammatory processes, disturbances in lipid metabolism, increased concentration of free fatty acids in blood/plasma, and increased fat accumulation in the body, therefore the risk of metabolic syndrome and type 2 diabetes increases (32, 33) and this finding was expected.

We found that the chances of diabetes in married, widowed, and divorced people compared to single people are 1.6, 3.2, and 1.4, respectively. Consistent with these results, Oraii et al. stated widows/divorced adults were at high risk for diabetes (6). Also, De Oliveira et al. showed marital status as a predictor of type 2 diabetes mellitus incidence (34). On the contrary, Mirzaei et al. said there was no significant relationship between marital status with undiagnosed or control of DM in patients (3). It seems in our context, the effective factors for this association should be further characterized. So, they may provide significant information in the better design and implementation of preventive programs.

Regarding the level of education, the present study's findings showed that illiterate people are at higher risk of diabetes, which was in line with the results of previous studies (27, 28, 35). Also, Hariri et al. pointed out that the level of education, mainly higher education, is a protective factor against diabetes and pre-diabetes (36). In contrast, some studies show that socioeconomic status, as measured by levels of education, occupation, or income, either they are not related (3, 6) or often are inversely related to diabetes (37–39). They argued that better-educated and wealthier people were more likely to become obese and thus more likely to develop diabetes. However, Oraii et al. reported higher levels of education were a determinant of better glycemic control among treated diabetic participants. Also, Najafipour et al. stated illiterate people had worse uncontrolled Diabetes Mellitus (5, 6). Considering that the population of illiterate people is more in our context, it seems that we should pay special attention to this variable in the planning and implementation of future interventions to prevent the occurrence of diabetes and its early diagnosis.

According to our results, there is no significant relationship between smoking and diabetes. In this regard, some studies also showed no association between ex-smokers and type 2 diabetes (40, 41). While, in another study, the risk of type 2 diabetes was significantly higher in active/inactive pre-diabetic smokers compared to active/inactive non-diabetic smokers (42). However, This discrepancy in the results may be due to the heterogeneous characteristics (sample size, age range, male-to-female ratio, and ethnicity) of the groups used in these studies. Therefore, the effect of smoking and opium consumption on diabetes in the Iranian population needs to be further investigated.

In this study, with increasing weight, the chance of developing diabetes increases by 1.009 times. On the other hand, the incidence of diabetes in tall people is reduced by 0.8 times compared to short people. The risk of developing diabetes at a BMI > 30 is about 2.5 times higher. In addition, the growth of wrist and waist circumference also increases the chances of developing diabetes. Also, people with an abnormal waist-to-hip ratio were 3.410 times more likely to develop diabetes. Almost most previous studies have confirmed such relationships (13, 15). Jayedi et al. based on a systematic review and dose-response meta-analysis of 216 cohort studies investigated anthropometric and adiposity indicators and the risk of type 2 diabetes. They reported that almost in all regions and ethnicities, BMI had a strong positive linear association with the risk of type 2 diabetes. Also, they found a larger waist circumference was strongly and linearly associated with a higher risk of type 2 diabetes. But they noted that there was an inverse association among hip circumference, waist circumference, and risk of type 2 diabetes for studies that controlled all of them. In contrast, they reported a positive association between hip circumference and the risk of type 2 diabetes for studies that did not control waist circumference (14). Future research should evaluate the association between body fat content in specific regions and the risk of diabetes risk in more detail.

Based on our results, increased sleep duration and a history of consuming sleeping pills were also associated with diabetes. Of course, based on the baseline data, the direction of this relationship is not clear, and it will be determined after the analysis of the follow-up data. There is strong evidence that diabetes causes sleep disturbances, but on the opposite side, the results of some previous studies showed that longer sleep duration is associated with an increased prevalence of DM and IGT (17, 43, 44). Although, based on a systematic review and meta-analysis study, it was found that short and long-term sleep duration contributed to the development of type 2 diabetes (43). Restriction of sleep reduces insulin sensitivity the next day by increasing cortisol levels in the nighttime (45, 46). In contrast, some infection markers such as IL-6, and C-reactive protein increase in people who report prolonged sleep, which accelerates the progression of diabetes and its complications (47, 48). Therefore, based on the results we obtained; it can be considered sleep duration/quality as a risk marker in monitoring diabetes. So more studies are needed to examine the definitive relationship.

In this study, a statistically significant relationship was also observed between physical activity and the incidence of diabetes. In such a way increasing the amount of physical activity reduces the chance of diabetes. Consistent with this result, Akbarzadeh et al. reported low levels of physical activity, in the modified model, were associated with a 38% increase in the risk of developing diabetes (28). Also, it is supported by other previous literature (20, 49). The results of a systematic review study show that inactivity leads to type 2 diabetes through decreased insulin sensitivity, gradual loss of beta cells, impaired glucose tolerance, and ultimately obesity (50). Physical activity has a protective effect on both people at risk for diabetes and people with diabetes (51). Although little is known about the type, intensity, and duration of beneficial physical activity in the region of our study, therefore, further studies should be conducted on the relationship between physical activity, its related factors, and the risk of developing diabetes.

### Research strengths and limitations

The large number, relative diversity, and heterogeneity of the study population are among the strengths of this study. As another advantage of the present work, DM has been defined based on FBS above 126 in addition to the use of anti-DM drugs. Through the PCS basic data analysis, there was an opportunity to examine the confounding effects of many potential confounders measured in highly qualified equipment and study environment.

The main limitation of the present study is its cross-sectional nature. Considering that most study participants were female and were limited to people over the age of 40, the results may be generalizable to a similar population.

## Conclusion

The results of this study showed that the prevalence of diabetes in Hoveyzeh city, Khuzestan, Iran, was almost high, and public health interventions and integrated management plans for earlier diagnosis, treatment, and better control of diabetes in this region are required. The important predictors of diabetes were increasing age, marital status, education level, obesity, increasing waist circumference, increasing BMI, and physical activity. Modifiable correlations of diabetes, including low education level, obesity, physical activity, and sleep quality emphasize the importance of public life improvement and effective preventive interventions, which should also be targeted at very young age groups. Due to the growing trend of elderly in Iran, paying attention to, caring for, and controlling diabetes since the third decade of life and health education for a healthy lifestyle are strongly recommended.

## Data availability statement

The original contributions presented in the study are included in the article/supplementary material, further inquiries can be directed to the corresponding author.

## Ethics statement

The studies involving human participants were reviewed and approved by the Research and Ethics Council of Ahvaz University of Medical Sciences (code: HCS-9902). The participants provided their written informed consent to participate in this study. No animal studies are presented in the manuscript.

## Author contributions

SH, SB, KZ, and KN were involved in designing this research. SH and KN collected the data. SB analyzed the data. KZ and SB were involved in the data interpretation and prepare the draft of the manuscript. KZ was responsible for writing and finalizing the manuscript. All authors read and approved the final version of the manuscript.
